# Pectoralis minor length measurements in three different scapula positions

**DOI:** 10.4102/sajp.v76i1.1487

**Published:** 2020-11-04

**Authors:** Muhle A. Komati, Francina E. Korkie, Piet Becker

**Affiliations:** 1 Department of Physiotherapy, Faculty of Health Sciences, University of Pretoria, Pretoria, South Africa; 2 Department of Biostatistics, Research Office, Faculty of Health Sciences, University of Pretoria, Pretoria, South Africa

**Keywords:** resting scapula, Pectoralis and Minor muscle, pectoralis minor index, Trapezius muscles, length measurements

## Abstract

**Background:**

The pectoralis minor (PM) muscle is commonly regarded as a contributor to abnormal scapula positioning. Subsequently, the muscle length of the scapular stabilising muscles may be affected, as these muscles assume a lengthened position, which over time causes weakness. There are inconsistencies regarding PM muscle length values because of the different techniques and positions used when the length of the PM muscle is measured.

**Objective:**

To determine the PM muscle length in participants aged 18−24 using a Vernier^®^ caliper and expressed as pectoralis minor index (PMI), with the scapula in three different positions.

**Method:**

The PM muscle length of 144 participants was measured with a Vernier^®^ caliper (intraclass correlation coefficient 0.83−0.87). Measurements were made with the scapula in the resting position, in an active and a passive posterior tilt position.

**Results:**

Significant differences were observed in PMI between the resting scapula position – 10.04 (confidence interval, CI 9.93–10.14) and active posterior tilt – 10.19 (CI 10.09–10.30) (*p* < 0.001); the resting position – 10.04 (CI 9.93–10.14) and passive posterior tilt – 10.77 (10.66–10.87) (*p* < 0.001) and active – 10.19 (CI 10.09–10.30) and passive posterior tilt 10.77 (10.66–10.87) (*p* < 0.001). The dominant side had lower PMI values than the non-dominant side.

**Conclusion:**

The significant differences between the active and posterior tilt positions suggested that optimal muscle length of PM was affected by the inner range strength of the lower fibres of Trapezius.

**Clinical implications:**

It is important that in clinical practice not only the length of PM in scapular misalignment but also the strength of the antagonistic muscles is considered.

## Introduction

Pectoralis minor (PM) muscle shortening has been attributed to sustained postures involving anterior tilting and protraction of the scapula (Borstad 2006; Rosa et al. 2016). The effect of an anteriorly tilted and protracted scapula on gleno-humeral function is threefold: the orientation of the glenoid to the humeral head is affected and may result in altered gleno-humeral arthrokinematics; the space between the acromion and humeral head is decreased and this may lead to compression of the sub-acromial structures and the scapula stabilising muscle may weaken as a result of the prolonged elongated position (Lee, Im & Kim 2020; Morais & Cruz 2016; Umehara et al. 2018). As PM is identified as a muscle that affects scapula positioning and gleno-humeral function, the effective measurement of PM length is important for rehabilitation purposes, to prevent and manage any upper limb dysfunction that may be caused by PM shortening.

There are discrepancies in the literature regarding the measurement of the PM muscle length, which include participant positioning and the position of the scapula when PM length is measured (Borstad & Ludewig 2005; Ko et al. 2016; Morais & Cruz 2016; Struyf et al. 2012). In these studies, participants were positioned either in standing or in supine (Borstad 2006, 2008; Borstad & Ludewig 2005; Cools et al. 2010; Finley et al. 2017; Ko et al. 2016; Lee et al. 2015; Mackenzie et al. 2015; Rosa et al. 2016, 2017; Struyf et al. 2012, 2014). In studies where PM length was measured in standing, the scapula could be in an anteriorly tilted position because of the influence of gravity on posture, thus demonstrating poor diagnostic accuracy and may provide inaccurate values for PM muscle length (Borstad 2006, 2008; Borstad & Ludewig 2005; Finley et al. 2017; Ko et al. 2016; Lee et al. 2015; Rosa et al. 2016, 2017).

Although the influences of gravity on posture are eliminated in supine and muscle relaxation in all surrounding muscles is optimised, the position of the arm with the elbow extended can affect the scapula position (Cools et al. 2010; Mackenzie et al. 2015; Struyf et al. 2012, 2014). With the elbow in extension, passive insufficiency of the long head of biceps brachii muscle may result in an anteriorly tilted scapula and therefore affect the PM muscle length (Kibler et al. 2013).

The position of the scapula is important when measuring the PM length. In several studies, the PM length is measured with the scapula in the resting position (Borstad 2006, 2008; Borstad & Ludewig 2005; Cools et al. 2010; Lee et al. 2015; Mackenzie et al. 2015; Rosa et al. 2016; Struyf et al. 2012, 2014). Rosa and colleagues (2017) argue that in the resting position of the scapula, the PM muscle is not in an optimal lengthened position and therefore added a measurement where the scapula is taken actively into a posteriorly tilted position. Finley and colleagues (2017) added a third position, where the scapula is taken passively into a full posteriorly tilted position. Significant differences were found in PM length when the three positions were compared (Finley et al. 2017). The participants were positioned in standing with the elbows extended in the three measurement positions. Standing as well as the elbows being extended does not favour the optimum PM length as previously mentioned. Therefore, the aim of our study was to determine the PM length with the scapula in three different positions in supine with the elbows flexed, eliminating the influences of gravity and of the biceps brachii muscle on PM length. We determined PM length, expressed as pectoralis minor index (PMI) in the resting, actively posteriorly tilted and passively posteriorly tilted positions of the scapula, using a Vernier^®^ caliper, for the dominant and non-dominant sides.

## Method

Our quantitative, observational, cross-sectional study was undertaken in the Department of Physiotherapy at the Prinshof Campus, Faculty of Health Sciences of Pretoria, South Africa. Non-probability convenience sampling was used. The population included student participants between the ages of 18 and 24 from the Faculty of Health Sciences, enrolled at the university Pretoria during the academic year 2018. Participants were recruited during academic contact time, and their appointments were scheduled at their convenience. The data collection was performed over a period of 9 days.

Participants were excluded if:

- they had previous fractures of the shoulder and/or shoulder girdle, as these injuries may have an influence on the shoulder girdle function and biomechanics (Levangie & Norkin 2001);
- they had a structural kyphosis and scoliosis because this type of kyphosis causes muscle imbalances and asymmetry in bony structure (Levangie & Norkin 2001);
- they had pain in the shoulders that interfered with activities of daily living (ADLs), as pain influences the function of the scapular stabilising muscles (Moezy, Sepehrifar & Dodaran 2014);
- they participated in elite sports as elite sport can cause anatomical adaptations in the gleno-humeral joint and muscles around the area (Hodgins et al. 2017).

### Sample size

To have 90% power to detect a statistically significant difference at a 0.05 level of significance, the target sample size was set at 128 participants. The target sample size was reached and surpassed by 16 participants, making the final number of participants, 144.

### Measures

The measurements were undertaken by the first and second authors as well as five undergraduate physiotherapy students (research assistants) who were familiar with these measurements. All the research assistants had a 1-h group training session, where they were prepared for their tasks for our study to ensure precision and competence. Their training was performed prior to a pilot study where all measurements were checked. The research assistants were assigned a specific task, and they performed the same task throughout our study to ensure good quality control. In addition, to ensure reliability and quality control, the second author palpated and marked all the origins and insertions of the PM muscle and the scapula landmarks.

Data were collected at five stations. Each station was separated by screens to respect the privacy of the participants. The participants were required to be in shorts and bikini top (if they were female) and bare-chested (if they were males). Pectoralis minor length is expressed as the PMI that is calculated as PM length (cm)/subject’s height × 100. This normalisation index is used to allow for soft tissue and body build variety (Borstad & Ludewig 2005).

#### Station 1 – Demographic information and marking of pectoralis minor landmarks

**FIGURE 1:**
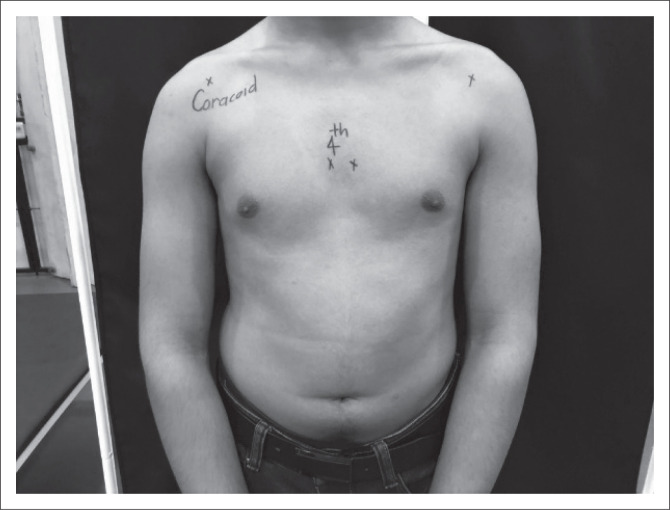
Pectoralis minor landmarks.

Prior to commencement, a demographic information sheet and consent form were completed by the student participants. Each participant was assigned a number that is mentioned on the demographic sheet. The participant’s number was then marked on the right scapula using a skin pencil. The landmarks for PM length were the medial inferior angle of the coracoid process and lateral to the sternocostal junction of the inferior aspect of the fourth rib. These landmarks have an intraclass correlation coefficient (ICC) of 0.96 (Borstad 2008) (Figure 1).

#### Station 2 – Postural analysis

A basic postural analysis was performed by the first research assistant following the prescribed plumb line (Kendall et al. 2005). The plumb line was made with a solid line drawn on a wall. Analysis focused on the general shoulder and scapula alignment, resting scapula position and thoracic posture. Postural analysis was performed to exclude any structural kyphosis. If a participant had a kyphosis, the participant was instructed to correct the kyphosis (extend the spine). The participants were classified as having a functional kyphosis and included in our study provided they could extend the spine. On analysis, when the shoulders and scapulae were anterior to the plumb line, the participants were categorised under a kyphotic posture. Participants were categorised under the ideal posture group when there was alignment from the earlobe to the greater trochanter (aligning well with the plumb line).

#### Station 3 – Exclusion of shoulder pain

The shoulder quadrant test was performed by the second research assistant to eliminate intra-articular shoulder pathology (Hengeveld & Banks 2015). If a participant was found to be present with a positive shoulder quadrant test, where shoulder pain interfered with their ADLs, they were excluded from our study and referred for further assessment and treatment by a physiotherapist.

#### Station 4 – Height measurement

The participants’ height was measured with a tape measure by the third research assistant. The participants stood with their backs against the wall, barefooted and their height was measured.

#### Station 5 – Pectoralis minor length test (pectoralis minor index)

**FIGURE 2:**
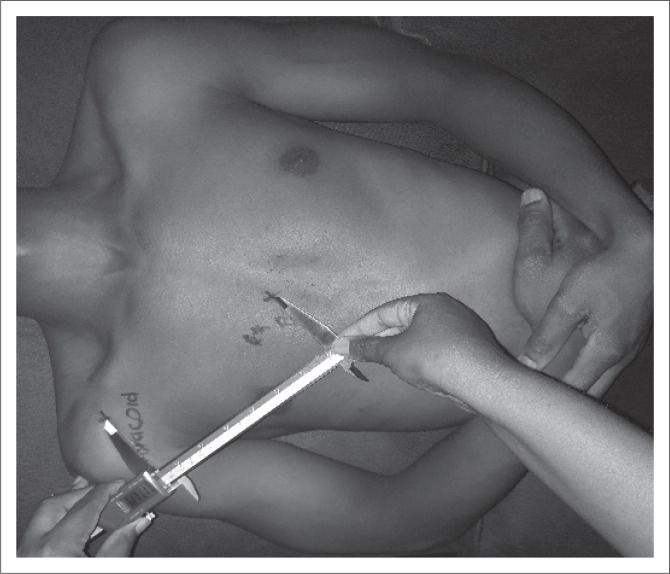
**Measurements using a** Vernier^®^ caliper.

Objective, controlled measurement of PM length was made by the first author using a standardised Vernier^®^ caliper, with an ICC of 0.83–0.87 (Borstad 2008:173) (Figure 2). The anatomical landmarks were used as measurement reference points. The length of the PM muscle was measured in three different positions of the scapula. In each position, the measurements were made thrice. The distances were read and captured by the fourth research assistant on a data capturing sheet. The Vernier^®^ caliper was turned upside down during measurements to prevent any bias of the first author, as only the fourth research assistant could see the readings on the caliper, and he or she documented the findings between measurements.

Participants were positioned in supine; both hands were placed on the abdomen with the shoulders slightly abducted and in a relaxed position of elbow flexion. The elbows were flexed to eliminate passive insufficiency of the biceps brachii muscle (Kibler et al. 2013; Lewis & Valentine 2007).

The first measurement was made in the resting position of the scapula, on both the right and the left sides. The participants were advised to completely relax the shoulder girdle, whilst the first author performed the measurements between the two anatomical points with a Vernier^®^ caliper.

The second position of measurement was made in an active posterior tilt position of the scapula. The participants were requested to posteriorly tilt their scapulae to full range of motion or to the point where they start to compensate with lumbar or thoracic extension. This active range of posterior titling indicated the active function or strength of the scapular stabilising muscles and in turn the active lengthening of PM. The distance between the two anatomical points was measured with a Vernier^®^ caliper on both the right and the left sides.

The final measurement was made at the end range of the scapula in a posterior tilt. This position was maintained by the fifth research assistant into a passive full range of the PM muscle length. The distance between the two anatomical points was measured with a Vernier^®^ caliper on both the right and the left sides.

## Data analysis

The data were coded and captured on an Excel spread sheet. The average of the three measurements in each position was used for analysis. Descriptive statistics were used to provide a brief summary of the mean PMI values on the dominant and non-dominant sides, in relation to the three testing positions. The data summary reports means, standard deviation (SD) and confidence interval (CI’s) of the PMI. Pectoralis minor index in respect to dominance was assessed in a linear mixed analysis, using a mixed-effects maximum likelihood regression. A significance level of *p* = 0.05 was set.

### Ethical consideration

Ethical approval was obtained from the committee of the University of Pretoria [114/2018] and informed consent was obtained from all participants prior to their participation in our study.

## Results

A total of 167 participants were evaluated, of whom 144 participants (consisting of 80 females and 64 males) were eligible for inclusion in our study. The remaining 23 participants were excluded because of intra-articular shoulder pain that interfered with ADLs (6), shoulder surgery (3), elite sports participation (11) and those falling outside the age category (3). In Table 1, the mean and SD of PMI for the dominant and non-dominant sides are presented for the three scapula positions. It is noted that the dominant side is shorter than the non-dominant side.

**TABLE 1:** Pectoralis minor index values for the dominant and non-dominant sides in the three testing positions of the scapula (*n* = 144).

Testing positions	PMI dominant		PMI non-dominant	
	Mean	SD	Mean	SD
Resting scapula	10.00	0.68	10.07	0.68
Active posterior tilt	10.16	0.66	10.23	0.68
Passive posterior tilt	10.74	0.71	10.79	0.71

PMI, Pectoralis minor index.

Table 2 presents the mean PMI values for the three scapula positions. Statistically significant differences were observed between the resting and active posterior tilt positions (*p* < 0.001), between the resting and passive posterior tilt positions (*p* < 0.001) and between the active and passive posterior tilt positions (*p* < 0.001).

**TABLE 2:** The mean pectoralis minor index values (left and right) obtained and compared in the three different scapula positions (*n* = 144).

	Resting scapula		Active posterior tilt		Passive posterior tilt		Difference when positions compared		*p*
	*N*	%	*N*	%	*N*	%	*N*	%	
Mean PMI (95% CI)	10.04	9.93–10.14	10.19	10.09–10.30	-	-	0.154	0.102–0.207	< 0.001
Mean PMI (95% CI)	10.04	9.93–10.14	-	-	10.77	10.66–10.87	0.728	0.675–0.781	< 0.001
Mean PMI (95% CI)	-	-	10.19	10.09–10.30	10.77	10.66–10.87	0.574	0.413–0.735	< 0.001

CI, confidence interval; PMI, pectoralis minor index.

## Discussion

The most important finding of our study is the statistically significant difference (*p* < 0.001) observed between the active and passive posterior tilt positions of the scapula. Finley et al. (2017) corroborate these findings. The difference between the active and passive tilt positions may indicate the role of the lower Trapezius (LT) not only on the scapula but also on the PM length. One of the more recent arguments in the evaluation of PM length is the role of the antagonist (LT) length and inner range strength on the resting position of the scapula (Kim, Lee & Yoo 2018; Morais & Cruz 2016). Adaptive shortening of PM is often seen as a result of a sustained postural position or occurs because of repetitive upper limb movements (Kendall et al. 2005; Kim et al. 2018). If shortened, the scapula is pulled into internal and downward rotation (Gutierrez-Espinoza et al. 2019; Kendall et al. 2005; Lee et al. 2018). In this downwardly rotated position LT is lengthened and may weaken, which raises the question of whether the adaptive shortening of PM is the cause or the result of the scapula position.

The effect of a shortened PM on scapular kinematics and upper limb function is well documented (Borstad & Ludewig 2005; Finley et al. 2017; Gutierrez-Espinoza et al. 2019; Kibler et al. 2013; Lee et al. 2018; Lewis & Valentine 2007). Is PM the only perpetrator responsible for the altered scapular position (Morais & Cruz 2016)? From the results of our study, one may argue that LT strength not only plays a role in scapular positioning but also affects the resting position of the scapula and therefore the resting length of PM. We also confirmed the principle that if a muscle is tested for length, the optimal muscle length (resting, active and passive posterior tilt of scapula) must be measured.

The PMI values show that the dominant side is shorter than the non-dominant side. Cools et al. (2010) and Struyf et al. (2014) reported similar results for PMI of the resting position of the scapula, with the dominant side having a significantly lower PMI than the non-dominant side. These findings may be because of the fact that the dominant side is stronger and most frequently used; therefore, it is more susceptible to have a shorter PM muscle length than the less frequently used non-dominant side (Kendall et al. 2005). The PMI values in the active posterior tilt position of the scapula of our study cannot be compared to other studies as no other study has compared the dominant and non-dominant sides in this position. Two studies that performed measurements with an active posterior tilt position of the scapula did not compare the dominant and non-dominant sides (Finley et al. 2017; Rosa et al. 2017). Similarly, the PMI values obtained in the passive posterior tilt position of the scapula in our study cannot be compared to other studies, as the only other study that performed measurements in the passive posterior tilt position again did not compare dominant and non-dominant sides (Finley 2017).

It is difficult to compare studies in terms of PMI values as the evaluation techniques are often different, and in most of the techniques biomechanical flaws are the main reason for the inconsistent outcomes that affect the validity and reliability (Morais & Cruz 2016). We addressed two of the main flaws in measurement: firstly, by limiting the effect of biceps brachii insufficiency by flexing the elbow, and with the hands placed on the abdomen. Secondly, not only was the resting length of PM measured, the effect of the antagonist was engaged in the active and passive posterior tilt positions. This may explain the differences we found compared to other studies.

The clinical contribution of our study is twofold. Firstly, the position of PM testing accounts for passive insufficiency of biceps brachii on the coracoid process and gravity is eliminated. Secondly, the significant difference between the active and passive posterior tilt positions confirms the influence of LT strength on PM length. A longitudinal study is recommended where the effect of LT strength on the scapula position as well as PM length is evaluated.

In our study, the strength of LT was not tested and this is noted as a limitation.

## Conclusion

The significant differences between the active and posterior tilt positions suggest that optimal muscle length of PM is affected by the inner range strength of the lower fibres of Trapezius. The PMI values show that the dominant side is shorter than the non-dominant side, with the dominant side having a significantly lower PMI than the non-dominant side.
